# Effect of Ammonium- and Phosphonium-Based Ionic Liquids on the Separation of Lactic Acid by Supported Ionic Liquid Membranes (SILMs)

**DOI:** 10.3390/membranes1020098

**Published:** 2011-05-13

**Authors:** Michiaki Matsumoto, Abhishek Panigrahi, Yuuki Murakami, Kazuo Kondo

**Affiliations:** 1 Department of Chemical Engineering and Materials Science, Doshisha University, Kyotanabe, Kyoto 610–0321, Japan; E-Mails: murakami19850801@yahoo.co.jp (Y.M.); kkondo@mail.doshisha.ac.jp (K.K.); 2 Department of Chemical Engineering, Indian Institute of Technology Guwahati, Guwahati, Assam 781039, India; E-Mail: a.panigrahi1989@gmail.com

**Keywords:** supported liquid membrane, ionic liquids, lactic acid, membrane separation

## Abstract

Biodegradable polymers have attracted much attention from an environmental point of view. Optically pure lactic acid that can be prepared by fermentation is one of the important raw materials for biodegradable polymer. The separation and purification of lactic acid from the fermentation broth are the major portions of the production costs. We proposed the application of supported ionic liquid membranes to recovering lactic acid. In this paper, the effect of ionic liquids, such as Aliquat 336, CYPHOS IL-101, CYPHOS IL-102, CYPHOS IL-104, CYPHOS IL-109 and CYPHOS IL-111 on the lactic acid permeation have been studied. Aliquat 336, CYPHOS IL-101 and CYPHOS IL-102 were found to be the best membrane solvents as far as membrane stability and permeation of lactic acid are concerned. CYPHOS IL-109 and CYPHOS IL-111 were found to be unsuitable, as they leak out from the pores of the supported liquid membrane (SLM), thereby allowing free transport of lactic acid as well as hydrochloric acid. CYPHOS IL-102 was found to be the most adequate (Permeation rate = 60.41%) among these ionic liquids as far as the separation of lactic acid is concerned. The permeation mechanisms, by which ionic liquid-water complexes act as the carrier of lactate and hydrochloric acid, were proposed. The experimental permeation results have been obtained as opposed to the expected values from the solution-diffusion mechanism.

## 1. Introduction

In supported liquid membrane (SLM), usually an organic liquid is embedded in small pores of a polymer support and is kept there by capillary forces. If the organic liquid is immiscible with the aqueous feed and strip streams, SLM can be used to separate the two aqueous phases. Since the volume of organic components used in the membrane is small and because the membrane separation is effectively a simultaneous extraction and re-extraction carried out in one technological step, it provides many advantages such as: expensive carriers may be used, higher separation factors are obtained, they are easy to scale-up, most importantly they have very low energy requirements, low capital and operating costs. Hence the use of liquid membrane technology for the separation of components is a promising technology [1].

Although SLMs have been widely studied for the separation and concentration of a variety of compounds and present many potential advantages over other separation methods, there have been very few large scale applications of SLM due to insufficient membrane stability. This problem can be due to the loss of the carrier and/or solvent from the membrane, which has an influence on both flux and selectivity [2]. If the organic liquid for the SLM can be chosen such that it remains stable within the capillaries of the porous support, then it can be used much more effectively.

Ionic liquids (ILs) are organic salts composed of organic cations and either organic or inorganic anions. The cation and anion can be varied to obtain an IL with the desired chemical and physical properties [3]. But normally, they remain in a liquid state over a wide range of temperatures, including room temperature. ILs are a new group of designer solvents of great interest, which have been recently widely studied as potential “green solvents”, especially in chemical and biochemical syntheses [4]. A great advantage of ILs is their practically negligible vapor pressure compared to widely used volatile organic solvents. Additionally, they have very high viscosities. These two key features of an IL enable them to be used as the liquid for SLMs [5,6,7,8,9,10,11,12].

The aim of this study is to find an IL that is suitable for use as a liquid in the SLM for the separation of lactic acid. Lactic acid is the raw material for biodegradable polymer (Poly lactic acid). Optically pure lactic acid can be prepared by fermentation. However, the majority of the production costs is accounted to the separation and purification of lactic acid from the fermentation broth. Therefore, current research is focused on effective, efficient and economical downstream processes for recovering lactic acid from fermentation broth. A number of processes for lactic acid recovery, such as solvent extraction, adsorption, direct distillation and electrodialysis, have been studied [13,14]. In our preliminary work, SLMs, based on ionic liquids such as Aliquat 336 and CYPHOS IL-101, were suggested to be suitable for lactic acid separation [15]. In this study, we examined the effect of ionic liquids, especially phosphonium ionic liquids, which have been less well studied [16,17,18], on the permeation behavior of lactic acid through SLM based on ionic liquids in detail.

Membrane separation of hydrophilic organic molecules by SLM based on ionic liquids has been considered to follow a permeation mechanism via formation of reversed micelles [16,19]. According to this mechanism, lactate ions form ionic liquid-lactate-water complexes at the membrane surface in contact with the feed side. These complexes including lactate ions are then transported across the membrane by their concentration gradient. At the membrane-permeate interface, these lactate ions are split and free ionic liquids molecules form the reversed micelles. This results in a net transport of lactate ions from feed side to permeate side.

## 2. Experimental Section

### 2.1. Chemicals

Hydrophilic porous polyvinylidene fluoride (PVDF) membranes (MILLIPORE, DURAPORE® MEMBRANE FILTERS, Millipore Corporation, Billerica, MA, USA, average micropore diameter 0.22 μm) were used as the support. CYPHOS IL-101, CYPHOS IL-102, CYPHOS IL-104, CYPHOS IL-109, CYPHOS IL-111, Aliquat 336 were the ionic liquids used. All the CYPHOS ionic liquids were purchased from STREM CHEMICALS, Newburyport, MA, USA. The molecular structure and properties of all these ionic liquids have been tabulated in Table 1. Concentrated hydrochloric acid and concentrated sodium hydroxide (Wako Pure Chemicals, Ltd., Osaka, Japan) were used for preparing the permeate solution and for adjusting pH of feed phase, respectively.

**Table 1:** Molecular structure and properties of the ionic liquids used in this study.

		**Viscosity^a^ cP**	**Water content^a^ wt%**
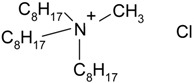	Aliquat 336	1450 (79.05) b	4.3 (20.3) b
	Trioctylmethylammonium chloride		
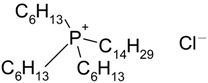	Cyphos IL-101	1824 (95.8) b	0.67 (12.8) b
	Trihexyltetradecylphosphonium chloride		
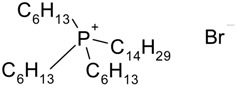	Cyphos IL-102	2094 (190.4) b	0.002 (6.1) b
	Trihexyltetradecylphosphonium bromide		
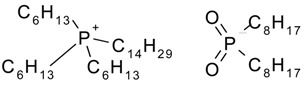	Cyphos IL-104	805.8	not measured
	Trihexyltetradecylphosphonium bis-2,4,4-trimethylpentylphosphinate		
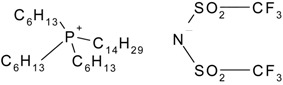	Cyphos IL-109	292.5	not measured
	Trihexyltetradecylphosphonium bis-trifluoromethylsulfonyl imide		
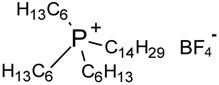	Cyphos IL-111	786.8	not measured
	Trihexyltetradecylphosphonium tetrafluoroborate		

a 25 °C;

b These values were determined using water saturated ionic liquid.

### 2.2. Preparation of the Supported Liquid Membrane based on Ionic Liquid

A PVDF membrane was used as the support to maintain the ionic liquid in the membrane. A hydrophilic PVDF membrane was soaked in a particular ionic liquid in a petri dish. These membranes were left in soaked state for 24 h. The membrane was then wiped and was dried in the vacuum desiccator for 24 h. After that the membrane was weighed. These membranes were used as the supported liquid membrane based on ionic liquid for the transport experiment.

### 2.3. Transport Experiment

**Figure 1:**
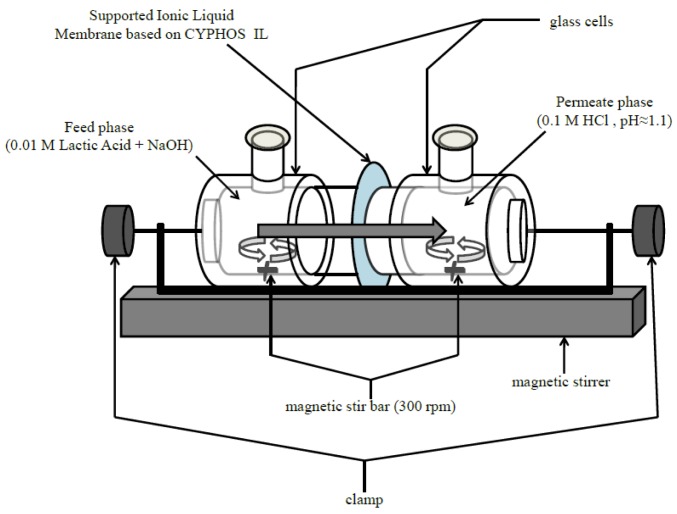
Schematic diagram of the experimental setup for the permeation experiment.

The apparatus consisted of two cells made of glass clamped together and placed on a magnetic stirrer as shown in Figure 1. The supported liquid membrane was put in between the two cells using vacuum grease (silicon grease) to avoid slipping and/or leakage.

The effective area (*A*) of this device is 12.5 cm^2^. The feed solution consisted of 0.01 M lactic acid with sodium hydroxide to maintain the initial pH approximately equal to 5.5. The permeate (receiving) phase was a 0.1 M aqueous solution of hydrochloric acid. The transport experiment for the SLM, based on the ionic liquid, was started by pouring 100 mL of feed and 100 mL of permeate solutions into their respective glass cells. A magnetic stir bar was used to constantly stir the feed and receiving solutions at 300 rpm throughout the experiment. The pH of feed side was checked using a pH meter (HORIBA) at intervals of 1 h. For the normal experiments (without pH control), the experiment was allowed to proceed freely without any intervention. For the pH control experiments, dilute sodium hydroxide solution was added to maintain the desired pH level, *i.e.*, 5.5. Thus, pH was monitored throughout the experiment at intervals of 2 h for transport experiments without pH control and at intervals of 1 h for transport experiments with pH control. The permeation experiment was stopped after 24 h and the membrane was weighed again.

The concentration of lactic acid in the feed and permeate phases were measured by drawing 0.5 mL samples at intervals of two h and using HPLC (Shimadzu LC-10ADvp) with an RI detector (Shimadzu RID-10A) to analyze the samples. Analysis was performed using a Shodex SUGAR SH-1011 (Showa Denko) column and 5 mM H_2_SO_4_ solution as the mobile phase.

### 2.4. Viscosity and Water Content Experiment

Because the viscosities of CYPHOS IL-101, CYPHOS IL-102 and Aliquat 336 when exposed to water were required, water-saturated ionic liquids were prepared. Their viscosities and water contents were measured by an Ostwald viscometer and Karl Fisher titration, respectively. Their measured results were also listed in Table 1.

### 2.5. Liquid–Liquid Extraction Experiment

Liquid–liquid extraction experiments were performed to determine the distribution of lactic acid in ionic liquid phase. 100 mL of 0.01 M lactic acid solution was prepared and its pH was adjusted close to 5.5 by adding NaOH. 0.5 mL of CYPHOS IL-101 and 0.5 mL of the lactic acid solution prepared as mentioned above was poured into the same safe lock tube. These safe lock tubes were shaken in a thermomixer (Eppendorf Thermomixer Comfort) for 2 h followed by centrifuging at 4000 rpm for ten minutes in a centrifuge (Eppendorf Centrifuge 5415 D). The mixture had been separated into two distinct phases. The aqueous phase in each safe lock tube was carefully transferred using a micropipette to a separate vial for HPLC analysis.

## 3. Results

### 3.1. Loss of IL from the SLM

The membrane weights were recorded before and after the transport experiment for the ionic liquids under study and tabulated in Table 2. The data from Table 2 indicates that Aliquat 336, CYPHOS IL-101 and CYPHOS IL-102 show hardly any loss of ionic liquid from the membrane pores over a period of 24 h. This is in agreement with the established fact that ionic liquids are not easily displaced from the pores under a cross-membrane pressure difference as they are immobilized by large van der Waals forces and due to their high viscosities. However, CYPHOS IL-104-based SLM showed a slight decrease in membrane weight and white streaks appeared on the membrane at the end of 24 h. CYPHOS IL-109 and CYPHOS IL-111 based SLMs showed a considerable loss of ionic liquid from the membrane and this was reflected in the decrease of membrane weight up to 30%. Since CYPHOS IL-104, -109 and -111 are less viscous, it is conceivable that the displacement of these ionic liquids from the membranes could be higher. Furthermore, they have more hydrophobic anion than the other ionic liquids. As is reported previously [20], the stability of SILM has been related with the hydrophobic/hydrophilic character of the ionic liquid, concluding that a better stabilization is obtained by using hydrophilic membranes support due to the hydrophilic nature of ionic liquids.

**Table 2:** Membrane weights before and after transport experiment.

**Ionic Liquid**	**Membrane weights [g] for experiment without pH control**		**Membrane experiment [g] for with pH control**	

	**Before**	**After**	**Before**	**After**
Aliquat 336	0.81	0.81	0.81	0.81
CYPHOS IL-101	0.83	0.83	0.83	0.82
CYPHOS IL-102	0.87	0.87	0.84	0.84
CYPHOS IL-104	0.83	0.82	0.81	0.79
CYPHOS IL-109	0.87	0.61	Failed	Failed
CYPHOS IL-111	0.83	0.78	0.84	0.77

### 3.2. Permeation Rates

The permeation rate (*P* [%]) was calculated by the following formula:

(1) $$P[\%]=\frac{C_{R,24h}}{C_{F,0}}\times 100$$

where *C*_R,24 h_ is concentration of lactic acid in receiving phase after 24 h and *C*_F,0_ is concentration of lactic acid in feed phase initially.

**Figure 2:**
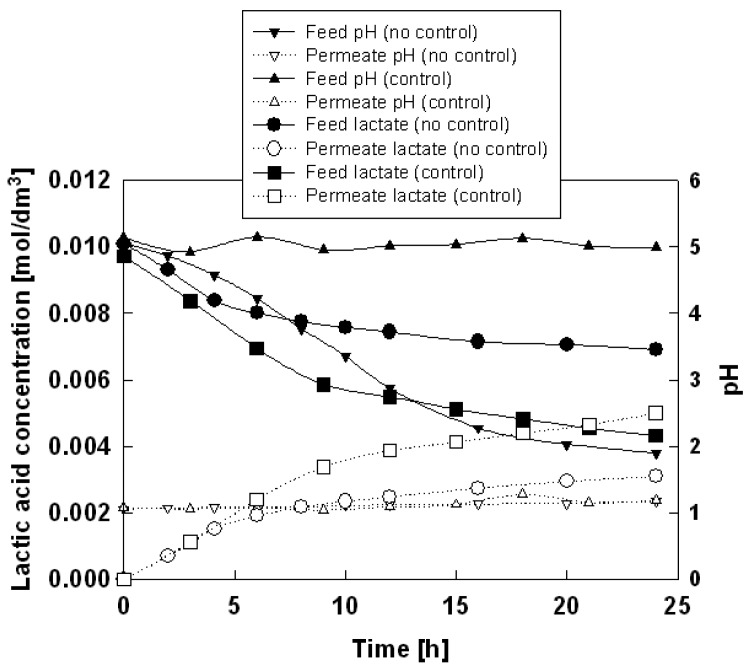
Time change of lactic acid concentration and pH for Ionic liquid Aliquat 336 in the absence and presence of pH control of feed phase.

The typical time courses of concentrations of lactic acid and pH in feed and permeate phases in the absence pH control are shown in Figure 2 using Aliquat 336 as an ionic liquid. A general observation is that the concentration of lactic acid in the feed phase decreases while that in the receiving phase increases with time. This is because lactic acid permeates through the membrane from the feed side into the permeate side. Another observation in the absence of pH control is that the pH of feed phase tends to decrease whereas the pH of the receiving phase does not change significantly over a period of 24 h. This decrease of pH on feed side is because hydrochloric acid permeates through the membrane from the permeate side into the feed side.

It is known that lactic acid, being a weak acid, dissociates to permeate species, lactate anion, at higher pH level. Therefore, to maintain the high pH of the feed phase, the experiments under the pH control of the feed phase was conducted and the result was also shown in Figure 2. The permeation rate was found to have improved as expected. The permeation rates of lactate through the SLM based on the ionic liquids in the absence and presence of pH control were summarized in Table 3, along with the pH value of feed phase after 24 h.

In the cases of transport experiment with SILM based on CYPHOS IL-101 and -102 without pH control, the pH remained above 4.1 in feed phase as such. In other words, a higher concentration of lactate ions was already available in the feed phase for diffusion across the membrane (p*K*_a_ of lactic acid = 3.86). Thus, even under pH control conditions, a significant increase in the permeation rate was not observed. CYPHOS IL-102-based SILM gave the highest permeation rate among all the ionic liquids studied. Lactate scarcely permeated through pure CYPHOS IL-104 membrane because CYPHOS IL-104 cannot extract lactate [21]. As shown later, it may be difficult to form CYPHOS IL-104–H_2_O complex as the acid carrier due to the hydrophobicity of the anion moiety. In the cases of CYPHOS IL-109 and -111, the experiments with pH control failed due to the leak of ionic liquids from the membrane.

**Table 3:** Permeation rates of lactate through SILMs with and without pH control, pH value of feed phase after 24 h and distribution ratio of lactic acid for the various ionic liquids.

**Ionic liquid**	**Permeation rate [%] (feed pH)**		**Distribution ratio of lactate**

	**no pH control**	**pH control**	
Aliquat 336	30.62 (1.894)	51.41	5.43
CYPHOS 101	48.91 (4.230)	58.78	2.50
CYPHOS 102	56.14 (4.105)	60.41	0.88
CYPHOS 104	15.08 (2.556)	14.7	0.01
CYPHOS 109	34.86 (1.476)	Failed	∼0
CYPHOS 111	28.36 (1.492)	Failed	0.08

## 4. Discussion

The permeation through SLMs is considered to follow the solution diffusion mechanism [22,23], where permeability, *P*, is the product of its distribution coeffcient, *K*, and diffusivity, *D*, in the membrane.

(2) P=D⋅K

The liquid-liquid extraction experiment was necessary to find out the distribution coefficient of the lactate ion in a particular ionic liquid. The distribution coefficients for lactate in ionic liquids used in this study are also shown in Table 3.

The above data can be summarized as follows:

Viscosity of water-saturated ionic liquid from Table 1:

(3) CYPHOS IL‐102>CYPHOS IL‐101>Aliquat336

Therefore, diffusivity of lactate ion increases in the following order because diffusivity varied inversely with the viscosity according to Wilke-Chang equation:

(4) CYPHOS IL‐102<CYPHOS IL‐101<Aliquat336

Distribution coefficient of lactate:

(5) CYPHOS IL‐102<CYPHOS IL‐101<Aliquat336

From the relations of Eqs.(4) and (5), expected permeability of lactate:

(6) CYPHOS IL‐102<CYPHOS IL‐101<Aliquat336

However, experimental results shown in Table 3 do not match the expected permeability order of Equation (6). Table 3 brings to light the fact that the actual permeation rates are in the reverse order as predicted by the expected permeability values.

According to the previous studies [16,19], the following steps have been suggested in the lactate (LA) transport through SLM based ionic liquid (IL).

(1) Splitting of the reverse micelles at the feed/SLM interface.
(2) Formation of IL–LA–H_2_O complexes.
(3) Transport of complexes through SLM.
(4) Splitting of the hydrated complexes on the SLM/permeate solution interface.
(5) Free IL molecules form reverse micelles (aggregates) at the permeate interface.
(6) Transport of the reverse micelles (aggregates) through SLM.

However, in this study through the permeation experiments the volumes of feed and permeate solutions were kept almost constant, suggesting that back transport of water was negligibly small. Alternatively, transport of hydrochloric acid from permeate to feed phases was observed. Therefore, we speculated that the IL–H_2_O complex play a role of acid carrier and IL–H_2_O complexes transport both lactate and hydrochloric acid in the reverse direction without waster transport. So, we propose the following steps.

(1) Formation of IL–LA–H_2_O complexes at the feed/SLM interface.
(2) Transport of lactate complexes through SLM.
(3) Splitting of the lactate complexes and formation of the IL–HCl–H_2_O complexes at the SLM/permeate solution interface.
(4) Transport of HCl complexes through SLM.
(5) Splitting of the HCl complexes at the feed/SLM interface.

In Equation (4), we estimated the diffusivities of the ionic liquids based on their viscosities. However, Table 1 shows that water contents in the ionic liquids increases in the following order.

(7) CYPHOS IL‐102<CYPHOS IL‐101<Aliquat336

This means that the size of the IL–H_2_O complexes considerably varies with the ionic liquids, suggesting that diffusivities of the IL–H_2_O complexes as acid carriers may vary with the ionic liquids. Further work is required to clear the detailed mechanism.

## 5. Conclusions

Ionic liquids are suitable membrane solvents for the preparation of supported liquid membranes because they are highly viscous and are not easily displaced from the pores of the PVDF support under a cross-membrane pressure gradient. They are very stable and have negligible vapor pressures at room temperature. Besides, the ionic liquids chosen for this study were immiscible in water. However, not all ionic liquids were found to be suitable. CYPHOS IL-104, -109 and -111 were unsuitable as membrane solvents for SILM for the separation of lactic acid. The CYPHOS IL-104 SILM gives very low permeation rate, whereas the CYPHOS IL-109 and -111 SILMs are unstable and a considerable loss of ionic liquid from the membrane was noted. The experimentally obtained results have been explained as opposed to the theoretically expected values. From the above analysis, it may be concluded that CYPHOS IL-102 followed by CYPHOS IL-101 are probably the best ionic liquids for separation of lactic acid on a lab-scale apparatus. However, because these ionic liquids are quite expensive at present, Aliquat 336, which gives quite good permeation rates for lactic acid, close to those of CYPHOS IL-101 and CYPHOS IL-102, may be more suitable for industrial applications.
